# Bacteriophages of Shiga Toxin-Producing *Escherichia coli* and Their Contribution to Pathogenicity

**DOI:** 10.3390/pathogens10040404

**Published:** 2021-03-29

**Authors:** Lorena Rodríguez-Rubio, Nadja Haarmann, Maike Schwidder, Maite Muniesa, Herbert Schmidt

**Affiliations:** 1 Department of Genetics, Microbiology and Statistics, University of Barcelona, Diagonal 643, 08028 Barcelona, Spain; lorenarodriguez@ub.edu (L.R.-R.); mmuniesa@ub.edu (M.M.); 2 Department of Food Microbiology and Hygiene, Institute of Food Science and Biotechnology, University of Hohenheim, 70599 Stuttgart, Germany; nadja.haarmann@uni-hohenheim.de (N.H.); maike.schwidder@uni-hohenheim.de (M.S.)

**Keywords:** Stx phages, STEC, Shiga toxins, lambdoid prophages, pathogenicity, virulence factors, antibiotic resistance genes

## Abstract

Shiga toxins (Stx) of Shiga toxin-producing *Escherichia coli* (STEC) are generally encoded in the genome of lambdoid bacteriophages, which spend the most time of their life cycle integrated as prophages in specific sites of the bacterial chromosome. Upon spontaneous induction or induction by chemical or physical stimuli, the *stx* genes are co-transcribed together with the late phase genes of the prophages. After being assembled in the cytoplasm, and after host cell lysis, mature bacteriophage particles are released into the environment, together with Stx. As members of the group of lambdoid phages, Stx phages share many genetic features with the archetypical temperate phage Lambda, but are heterogeneous in their DNA sequences due to frequent recombination events. In addition to Stx phages, the genome of pathogenic STEC bacteria may contain numerous prophages, which are either cryptic or functional. These prophages may carry foreign genes, some of them related to virulence, besides those necessary for the phage life cycle. Since the production of one or more Stx is considered the major pathogenicity factor of STEC, we aim to highlight the new insights on the contribution of Stx phages and other STEC phages to pathogenicity.

## 1. Introduction

Soon after the first reported outbreak with pathogenic Shiga toxin-producing *E. coli* (STEC) O157:H7 (syn. enterohemorrhagic *E. coli* (EHEC)) in Oregon and Michigan, USA, in 1983, the ability of these pathogens to produce Stx (syn. Shiga-like toxin, verocytotoxin, verotoxin) was demonstrated to be encoded by bacteriophages [1]. Following this observation, Alison O’Brien’s group genetically and morphologically characterized two Stx converting phages induced from *E. coli* O26 and *E. coli* O157:H7 strains [2]. Phages H19J and 933J showed a typical head-tail structure with short tails. Some years later, Huang et al. demonstrated the homology of Stx1-converting bacteriophage H19-B to phage lambda by southern blot hybridization and restriction analysis [3]. During the following years, methodological developments allowed for an accurate characterization of Stx phages, making it clear that these phages comprise a family of genetically heterogeneous members [4,5,6,7,8,9]. Whole genome sequencing yielded the sequence data of hundreds of pathogenic STEC genomes in the National Center for Biotechnology Information (NCBI) database (https://www.ncbi.nlm.nih.gov/genome/browse#!/prokaryotes/167/, accessed on 26 March 2021), which confirmed the heterogeneity of Stx phage genomes. These differences, in turn, influence the bacterial genome structure and its functionality [10]. Furthermore, the prophage sequences demonstrate that all Stx phages conserve a basic lambdoid structure that is discussed below.

## 2. Morphology, Genetic Structure and Integration Sites of Stx Phages

All known Stx phages are double-stranded DNA-phages with a functional genetic organization similar to that of the archetypical phage lambda, which is one of the best studied *E*. *coli* phages [11,12]. Stx phages share a common head-tail structure ranging from icosahedral or elongated heads to contractile, non-contractile, or short tails with or without tail fibers [9,13,14,15,16,17,18,19].

Although all Stx phages share the same lambda-like genetic structure, significant variations in their genetic composition occur and genome sizes ranging from 28.7 to 71.9 kb have been described [17,20,21,22].

According to their morphological structure, Stx phages are classified into different families of the order *Caudovirales*. For example, the Stx2a phage 933W of the *E. coli* O157:H7 strain EDL933 consists of a short tail and regular hexagonal head and belongs to the *Podoviridae* family [18], whereas the prototype Stx1 phage H19-B [3,15] consists of an elongated head with a long non-contractile tail compatible with phages of the *Siphoviridae* family. While most of the characterized Stx2 phages belong to the family *Podoviridae* [9,23,24], only a few reports exist where Stx phages have been described as members of the family *Myoviridae* [25].

A large majority of short-tailed Stx phages (among them the Stx2 phages 933W and Sp5 of *E. coli* O157 strains EDL933 and Sakai) use highly conserved tail spike proteins for host recognition [23]. Sequence homologues of the tail spike protein gene of short-tailed Stx phages were also found in the genomes of Stx phages of the *Siphoviridae* and *Myoviridae* family [26]. Essential for phage adsorption is the highly conserved receptor protein YaeT (also known as BamA) on the bacterial cell surface and its orthologues, which ensure the spread among various members of the family of *Enterobacteriaceae* [23,27]. Two other potential receptor proteins, FadL and LamB, have been described for phages Stx2Φ-I and Stx2Φ-II, isolated from clinical STEC strains [28], but they could not be confirmed in later studies as functional receptors for phage Sp5 of the *E. coli* O157 strain Sakai [27].

**Figure 1:**
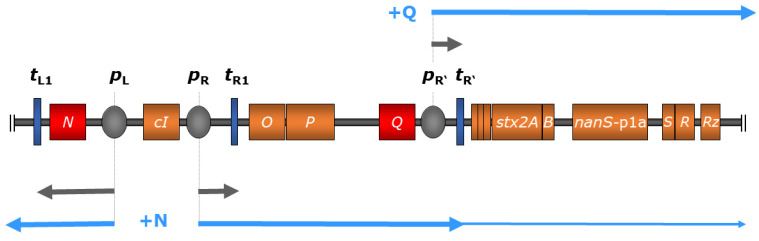
Regulation scheme of *stx* expression in bacteriophage 933W of *E. coli* O157:H7 strain EDL 933 comprising relevant genes (colored in orange) and regulatory elements (not to scale), modified from Wagner and Waldor 2002 [30]. During the lysogenic state (indicated in grey arrows), transcription is inhibited through binding of the *c*I-encoded repressor protein to operator sites of the early promoters *p*_L_ and *p*_R_ (colored in grey); transcription is also terminated by downstream terminators (dark blue). Upon phage induction, autocleavage of the *cI*-encoded repressor protein allows transcription at *p*_L_, resulting in the production of phage-encoded antiterminator protein N (red), which enables polymerase read-through at downstream terminators including *t*_L1_ and *t*_R1_. This, in turn, leads to the expression of the late-phase antiterminator Q (red), which facilitates transcription initiating at the late-phase promoter *p*_R’_, transcending terminator *t*_R’_, and resulting in the expression of downstream genes including *stx* (indicated in light blue arrows). Additionally, the expression of *O*- and *P*-encoded phage replication products leads to increased Stx production by amplifying *stx* copy numbers [30,39].

As lambdoid phages, Stx phages share a general genetic structure with immediate early, delayed early, and late phase genes. All of them possess a common regulatory system that includes different variants of *cro*, *cI*, *N*, and *Q* genes, which are involved in the regulation of the phage entering the lysogenic or lytic life cycle [19,20,29,30]. All Stx phages known so far show a conserved location of the *stx* genes in the late regulatory region of the prophage genomes (Figure 1) [8,11,19,31,32]. More precisely, *stx* genes are located downstream of the gene encoding the antiterminator protein Q and upstream of the lysis cassette consisting of *S*, *R*, and *Rz*, and are thereby under control of the late promoter *p*_R’_ [7,8,18,19,33] (Figure 1). However, the genomic region between the antiterminator Q gene and *stx* has been shown to be diverse among Stx phages of the same subtype, and is therefore supposed to have an influence on the Stx expression level [34]. Variations of the *Q* gene have also been reported, which are thought to have a minor impact on Stx expression and correlate loosely with a clinical or bovine origin of the strains [35]. It is hypothesized that *Q_21_* genes similar to the one of φ21 are often associated with lower Stx expression than the *Q_933_* gene variants of phage 933W [36,37]. Nevertheless, these results are not completely clear and could not be supported by other studies [38]. Most probably, there are various unidentified factors also contributing to the level of Stx expression [35,38].

Genomic differences have also been reported for the early regulatory regions of Stx phages. For example, Stx2 phage 933W contains three operator sites in the right operator region, but only two operator sites in the left operator region, which is different from phage lambda and most other lambdoid phages [39]. In contrast, Stx1 phage H-19B contains four operator sites in the right operator region [40]. It is not well understood how these differences in the early regulatory region affect repressor/operator interactions and, thereby, expression of Stx. However, it was demonstrated that spontaneous induction occurs more readily in Stx phages than in lambdoid prophages without *stx* genes [39,41].

During the lysogenic state, transcription of most phage genes is mostly silenced by the CI repressor binding at the operators within the early regulatory region [42]. Although expression of certain Stx phage genes during the lysogenic state has been reported [43], it was attributed to a small subset of cells that spontaneously induced the lytic cycle. Thereby, the transcription of phage genes is terminated at *t*_R’_ located directly downstream of *p*_R’_, thus preventing the transcription of *stx* genes. Upon phage induction, a cascade of regulatory events leads to the expression of early and late antiterminator proteins N and Q, respectively, allowing polymerase read-through of downstream terminators [30] (Figure 1).

Interestingly, a continuous transcription activity at phage late promoter *p*_R’_, which is terminated directly downstream at *t*_R’_, generates a short RNA byproduct under lysogenic conditions [44]. It was demonstrated that this regulatory small RNA represses expression of Stx1 under lysogenic conditions and modulates host fitness [45].

Stx phages can harbor a number of additional genes acquired by horizontal gene transfer [9,20]. These so-called morons (“more-on” refers to additional DNA on the phage genomes) are mainly found in the late phage region and usually have a different nucleotide composition compared to the rest of the phage genome. Furthermore, morons may have their own promoter and terminator sequences, so the transcription is independent from phage induction. These genes have no obvious function for the phage but are typically beneficial for the host [9,46].

The STEC genome can contain various Stx prophages and diverse non-Stx prophages [47]. Several strains naturally carry more than one Stx phage and double, or even triple, lysogens of the same Stx phage can be experimentally produced [48,49]. Stx phage integrases seem to have evolved to recognize different insertion sites within the bacterial chromosome. Thus, although each Stx phage integrates preferentially in one particular site, the integrase is able to recognize secondary sites for the phage genome integration if this preferred site is occupied or deleted [50].

Several chromosomal insertion sites have been described for Stx phages: *yehV* encoding a regulator for curli expression [51,52], *wrbA* encoding the Trp repressor-binding protein [18], *yecE* whose function is unknown [32], *sbcB* encoding an exonuclease [17,53], Z2577 encoding an oxidoreductase [54], *ssrA* encoding a tmRNA [55], *prfC* encoding a peptide chain release factor [56], *argW* encoding a tRNA [57], and the intergenic region between *torS* and *torT* genes [56]. In addition, a study by Steyert et al. revealed five novel insertion sites (*potC*, *yciD*, *ynfH*, *serU*, *yjbM*) in Locus of Enterocyte Effacement (LEE)-negative STEC isolates that had not been reported to be occupied by Stx phages before [58]. Several new insertion sites have been described for Stx phages carrying the novel Stx2 subtypes Stx2h and Stx2k [21,59]. The Stx2k prophage was found to be integrated adjacent to the *yjjG*, encoding a nucleotide phosphatase [59,60]. Different insertion sites were described for the Stx2k phages including *dusA*, which encodes a tRNA dihydroxyuridine synthase A [61], *yccA*, a predicted transmembrane protein [62,63], and the *zur* gene encoding a zinc uptake regulator [21,64].

Unlike phage lambda, Stx phages can occur as multiple isogenic prophages in the bacterial chromosome at different insertion sites [50,65]. Whereas phage lambda leads to host immunity, Stx phages are able to evade superinfection immunity [48,49,66]. For example, Stx2 phage Φ24_B_ was shown to integrate into a single host at least three times and furthermore, it was demonstrated that the frequency of multiple lysogens increased with each integrated prophage [9,67]. Different results were reported concerning the influence of multiple lysogens on the toxin expression level: interestingly, experiments with a double isogenic Stx2 phage showed an enhanced production level of Stx [65], whereas other studies with two different Stx2 prophages showed reduced toxin levels [48,68].

## 3. Induction, Expression and Release of Stx

When Stx phages choose the lysogenic pathway, phage DNA is inserted into the *E. coli* chromosome, forming a prophage that is replicated together with the bacterial chromosome, transferred to the bacterial progeny by vertical gene transfer and maintained for many cell generations. When diverse environmental conditions threaten the viability of the bacterial cell, these stimuli trigger the SOS response, activating the induction of the prophage. Several of these stimuli have been identified including changes in pH, particularly low pH [69], presence of iron [70], presence (or absence) of ions, which also confers a role on chelating agents such as EDTA and sodium citrate [71,72], several antibiotics including growth promoters [73,74], and other agents causing DNA damage such as mitomycin C or hydrogen peroxide [75,76,77].

After induction, prophages are excised from the chromosome. The viral DNA, which exists as a separate molecule within the bacterial cell, then replicates separately from the host bacterial DNA as an extrachromosomal element [78]. It has been found that *stx* can be detected in a circular, extrachromosomal state when the non-chromosomal elements are analyzed by southern blot after a PFGE of S1-digested DNA from STEC strains [79]. Moreover, circularized plasmid-like pseudolysogens of Stx phages have been observed in studies of integration of Stx phage Φ24B [67]. Plasmids derived from Stx phages have also been used to study the efficiency of DNA replication of lambdoid phages [78].

During replication, expression of the phage structural proteins and Stx takes place. The structural components are assembled into new Stx phage particles, which are released from the cell by the action of phage lytic proteins expressed at the end of the induction process. These proteins cause the disruption of the bacterial host cell, allowing the release and spread of Stx [8,42], which is the main virulence factor determining the severity and lethality of the STEC infection [80].

Stx can also be released by outer membrane vesicles (OMV) [81,82,83,84]. These OMVs protect Stx and other proteins from degradation by proteases and mask its presence in cytotoxicity or bead-enzyme-linked immunosorbent assays [81]. It was shown that OMVs from the hypervirulent O104:H4 outbreak strain are also internalized by intestinal epithelial cells despite not expressing the typical GB_3_ receptor [84]. A major study by Bielaszewska et al. also described this internalization strategy. Briefly, vesicles were taken up via dynamin-dependent endocytosis, followed by retrograde transport of the Stx holotoxin in early endosomes toward the Golgi complex and endoplasmatic reticulum. The enzymatic active Stx2A subunit could then be transported to the cytosol and bind to the ribosome [83].

In addition to Stx release, the new Stx phages are set free, which allows the dissemination and acquisition of the *stx* gene among susceptible cells (*E. coli* or even other genera) present in the same biome [85], contributing to the evolution of STEC [86]. In this context, *stx* genes have been detected in *Citrobacter freundii* [87], *Enterobacter cloacae* [88], *Shigella sonnei* [89], and *Aeromonas* spp. [90].

The effective production of Stx2 is always dependent on phage induction, whereas Stx release is dependent on cell lysis [42]. However, a different situation can be observed for Stx1, encoded by Stx1 phages [58,70,91]. The expression of Stx1 is caused by two independent promoters. The first is a late phage promoter *p*_R’_ dependent on phage induction (as for Stx2 phages), which allows the expression and release of the toxin by the phage-mediated cell lysis. The second is a specific Stx1 promoter containing a binding site for Fur protein, which makes complexes with iron. Thus, in the presence of iron, Fur blocks Stx1 expression, while in the absence of iron, this repression does not occur and Stx1 is expressed. This situation is entirely independent of phage induction, and Stx1 levels of production are similar to those observed under conditions where the Stx1 phage is not induced [70]. The main consequence of the phage-independent expression of Stx1 is that cells expressing Stx1 can avoid cell lysis, enhancing their survival. Fewer strains producing Stx1 phages means a lower occurrence of free Stx1 phages compared with Stx2 phages, which has been confirmed by analyzing free Stx1 vs. Stx2 phages in extracellular biomes [91].

In any case, Stx2 or Stx1 phage induction poses a serious threat for the survival of the STEC population, which must sacrifice its prevalence for the sake of increasing its virulence. The solution of the paradox presented by Stx as a virulence factor that forces phage activation and cell lysis in order to be expressed and released, is obtained when considering the heterogeneity of the STEC population. In a bacterial population, not all bacterial cells behave synchronously since they are not in the same physiological or growth state, therefore not all of them activate phage induction simultaneously. Thus, one subpopulation will induce Stx phages, producing new virions and expressing the toxin, while another subpopulation remains in the lysogenic state, enhancing its survival and becoming the population’s reservoir [92]. Although the mechanism dealing with the differences between the inducible and the non-inducible stage have not yet been completely elucidated, the growth state seems to play a role. Cells reaching the stationary phase prevent induction better than cells in the exponential phase. The RpoS factor, highly expressed in *E. coli* cells in the stationary phase, was shown to cause a dramatic delay in Stx phage induction within the *E. coli* population, and overexpression of RpoS resulted in a large number of *E. coli* cells that do not induce the Stx prophage [92]. In contrast, in *E. coli*, lambda prophage induction has been shown to be regulated by the OxyR protein [93].

The differential induction of Stx phages within the STEC population is indeed considered an altruistic strategy shown by a fraction of the STEC cells, rendering the expression of Stx a positive force for the benefit of the whole population [94]. It has been seen in cells spontaneously inducing Stx phages [41] under natural conditions but also in the presence of H_2_O_2_, which is produced by neutrophiles during STEC infection in the human body [94].

## 4. Stx Phages as Pathogenic Principle

In addition to *stx*, many additional genes have been described in Stx prophage genomes, which may contribute to pathogenicity and virulence, but also to the competitiveness with other gut bacteria in the human host. There are a number of reviews and book chapters that have described the role of some genes in the Stx phages that contribute to regulating pathogenicity in STEC [9,10,46,71,95], and therefore, their structure, function, and roles in pathogenicity will not be reviewed here.

**Figure 2:**
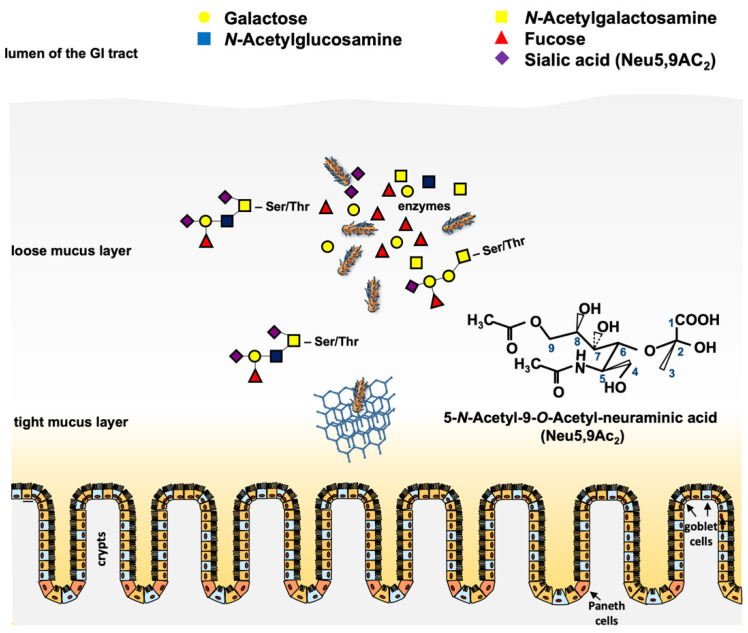
Scheme of putative functions of the phage-encoded *O*-acetyl esterase in the large intestine. Pathogenic STEC cells have to traverse the loose and the tight mucus layer to reach the epithelium for adherence and colonization. Mucinases and other proteases play a role in that process. Cleavage of *O*-acteyl residues from terminal *O*-glycans (e.g., Neu5,9Ac_2_) by chromosomal and phage-encoded *O*-acetyl esterases results in deacetylated free sialic acids such as *N*-acetyl neuraminic acid, which can be metabolized by the bacteria [113]. The chemical structure of Neu5,9Ac_2_ is shown. Honeycomb structure = mucin network. Paneth cells and goblet cells are indicated.

However, there is one newer gene family that is worth describing, since it is present in a number of Stx and non-Stx phages of pathogenic STEC. In preliminary work, an open reading frame (ORF) located downstream of the *stx* operon in the genome of phage 933W of *E. coli* O157:H7 and other relevant STEC serotypes was identified [96]. This ORF (z1466) could be induced in microarray experiments together with *stx* upon norfloxacin treatment of *E. coli* O157:H7 strain EDL933 [97]. When cultured in simulated colonic environmental medium (SCEM), a 40-fold expression of the corresponding protein P42 was observed [98]. Comparative analyses showed that the gene z1466 is highly homologous to a Neu5,9Ac_2_-esterase gene from *E. coli* that has already been in the focus of several studies [99,100]. By molecular and biochemical analyses, it was shown that z1466 indeed encodes a Neu5,9Ac_2_-acetylesterase, with an active esterase function similar to the chromosomally-encoded NanS, present in many *E. coli* strains [101]. Moreover, the gene was significantly longer than *nanS* and contained regions without homology to any known genes [102]. The function of the esterase as well as the role of seven vs. 10 Neu5,9Ac_2_ acetylesterases (NanS-p) from *E. coli* O157:H7 strain EDL933, and of five NanS-ps from *E. coli* O104:H4 strains C227-11φcu were analyzed, and it was shown that all these enzymes were encoded in prophage genomes that produced active esterases from their corresponding *nanS*-p alleles [101,103]. These results were in concordance with Eric Vimr’s early work [99] showing that cleavage of the *O*-acetyl residues from Neu5,9Ac_2_ allowed the lysogen to grow with Neu5,9Ac_2_ as a single carbon source. Furthermore, experiments with bovine maxillary gland mucin revealed the cleavage of mono, di, and triacetylated *O*-glycans by the NanS-p enzymes [102]. Similar experiments with the 2011 outbreak strain O104:H4 C227-11φcu revealed comparable results [103]. Taken together, the experiments have shown that these phage-encoded NanS-p enzymes can be used by pathogenic STEC strains to utilize mucin components for their growth, conferring an advantage to the lysogens [100,102,103,104] (Figure 2).

The fact that *nanS-p* genes are generally located in phage genomes and that Neu5,9-*O*-acteylesterases are able to cleave *O*-acetyl residues from sugar moieties [105] raises the question whether this enzyme may play a role in the phage replication cycle itself and consequently could contribute to the STEC infection process. A very interesting aspect of the NanS-p function came from the structural annotation by homology modeling of the esterase domain and crystal structure analysis of the C-terminal domain of the conserved carbohydrate esterase vb_24B_21 from the Stx phage φ24_B_, which is homologous to *nanS*-p [104]. The authors proposed a lectin-like, jelly-roll sandwich-fold in the C-terminus with a proposed function in carbohydrate-binding for this domain [104]. It was hypothesized that such a structure could target the enzyme to its substrate to increase the local concentration and to improve catalysis, as shown for similar enzymes [106,107]. Up to now, there is no experimental evidence that this is the case for NanS-ps of pathogenic *E. coli.* However, carbohydrate-binding may be advantageous for pathogenic *E. coli,* which can use mucins with a particular carbohydrate structure as the substrate.

Another possibility is that NanS-ps could also be an advantage for the phages itself by enhancing the recognition of phage receptors at the bacterial outer membrane surface. In Gram-negative bacteria, phages have to encounter the LPS, which may function as an initial binding site for infection [108,109,110]. *O*-antigens of the lipopolysaccharide may be acetylated, and cleavage of these *O*-acetyl groups may facilitate phage binding [109,111] as well as subsequent traversing of the LPS to reach the specific receptor sites located at the outer membrane [112]. Whether NanS-ps may play a role for the attachment of Stx phages remains to be elucidated.

## 5. New Stx Phages

Aside from the two main immunologically distinct toxin types Stx1 and Stx2 [114], several subtypes have been described according to the nomenclature proposed by Scheutz et al. [115]. Whereas Stx1 presents the more homogeneous group consisting of subtypes Stx1a, Stx1c, and Stx1d, the Stx2 group is more heterogeneous and also more frequently associated with severe forms of diseases such as hemorrhagic colitis or HUS [116,117]. Additionally, the level of Stx expression has been shown to be correlated with different Stx subtypes and phages [118]. In a study by Fitzgerald et al., using an *E. coli* O157 strain harboring both Stx2a and Stx2c phages, it was demonstrated that Stx2a was induced more rapidly and to higher levels than Stx2c [119]. Whereas Stx2c phages seem to be highly homogeneous, as reported by Ogura et al., during a comprehensive analysis of Stx2 phages in 123 EHEC O157 strains, Stx2a phages could further be subtyped according to their replication proteins. The respective Stx2a subtypes also correlated with the level of Stx2a expression in the host strains [68].

In addition to the well-known subtypes Stx2a, Stx2b, Stx2c, Stx2d, Stx2e, Stx2f, and Stx2g, several phages harboring new *stx* subtypes were described. For example, the novel Stx2 subtype h, which was found in STEC strains isolated from intestinal tracts of healthy marmots in China. The Stx2h prophage was reported to be 49,713 bp in size [59]. Sequence analysis revealed 93 predicted coding sequences (CDSs), out of which 37 were hypothetical proteins or mobile elements with unknown function, while phage-specific genes, encoding proteins responsible for integration, transcriptional regulation, and lysis, were found in accordance to other Stx2 phages [59].

A further Stx2 subtype, Stx2i, was described in STEC isolates recovered from shrimps and bivalves, but no further information concerning the genomic characteristics of the respective phages was given [120,121]. The same applies to the subtype Stx2j, which was mentioned in a publication by Yang et al., but without further information [21]. The latest subtype described so far, Stx2k, was identified in *E. coli* strains isolated from different sources in China including humans, animals, and raw meat [21]. Interestingly, the isolated *E. coli* strains, which carried the Stx2k phage, showed considerable heterogeneity in serotype, genome sequence, and virulence gene profile. One of the analyzed STEC strains even harbored the plasmid-encoded heat-stable enterotoxin gene *sta* as well as two copies of enterotoxin gene *stb*, which were located on the chromosome. As the presence of these enterotoxins is characteristic for enterotoxigenic *E. coli* (ETEC), they reveal an STEC/ETEC hybrid pathotype and point out the contribution of phages to the rise of new virulent bacteria. Similar results were found for the Stx2k-converting phages of these strains as they also showed considerable heterogeneity concerning insertion sites, genetic content, and structure as well as in *stx* expression level and cytotoxicity. The phage genome sizes ranged from 28,694 bp to 54,005 bp, with predicted CDSs between 53 and 86.

## 6. Evolutionary Viewpoints

Although it is suggested that bacteriophages may play a major role in the development of pathogenic STEC-mediated disease, general questions on phage ecology remain unresolved. Since *stx* genes have never been found on plasmids or in the chromosome in nature during the last 30 years of STEC research, the question arises whether bacteria or phages benefit from the mobile *stx* genes or whether both of them obtain the benefit. In some studies, it has been shown that Stx production might confer protection to bacteria against predation by protozoans in its ecological niche [122], providing a plausible biological explanation for the wide distribution of *stx* in gut bacteria such as *E. coli*. Stx-producing bacteria killed *Tetrahymena thermophila* when grown in co-culture, and treatment with purified Stx also caused the death of protozoans [122,123]. Moreover, the bacterial SOS response system that is involved in Stx phage induction was also involved in that process. The findings of this study were in concordance with earlier observations showing that the Stx prophage enhanced the fitness of *E. coli* lysogens and wildtype *E. coli* O157:H7 strains in the presence of *Tetrahymena pyriformis,* and it was suggested that most of the advantages were related to Stx production [123]. Protozoa are widely distributed in nature and were present on Earth long before human beings. The fact that Stx-producing bacteria occur in many distinct ecological niches indicates that the primary role of Stx may not be causing disease in humans, but protecting the bacterial strains from predation [123]. However, it should be mentioned that similar effects on *Paramecium caudatum* and *T. pyriformis* could not be confirmed by other authors [124] and, therefore, the question of the biological function of Stx still remains open. However, to properly evaluate the effect of Stx phages on STEC survival in a bovine host, the impact of Stx on the bovine eukaryotic cells and their immunological system should also be considered, as is further discussed in Section 8.

## 7. Influence of Stx Phages on the Bacterial Transcriptome

**Figure 3:**
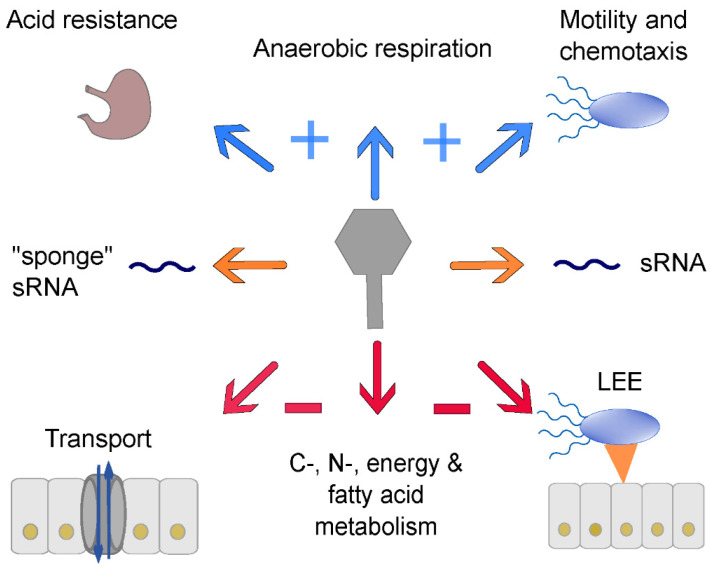
Influence of Stx prophages on the bacterial host transcriptome; blue arrows indicate upregulation of genes for acid resistance, anaerobic respiration, and motility and chemotaxis; orange arrows point to transcriptomic changes via sRNAs; red arrows symbolize downregulation of transport, C-, N-, energy, and fatty acid metabolism, and LEE genes. Details are described in the text.

In numerous studies, Stx prophages have been shown to influence their host strains not only by providing genes for new enzymes, toxins, etc., but also by changing their transcription patterns in many different metabolic categories, examples of which will be discussed below [44,97,125,126,127,128,129] (Figure 3). Early experiments were performed with microarrays containing oligonucleotides of *E. coli* strains EDL933 and RIMD0509952 as well as *E. coli* K-12 strain MG1655 [97] to discern transcriptomic changes following norfloxacin treatment. It could be shown that most of the upregulated genes were phage-related genes. Among these, the most strongly upregulated genes were the late phage genes (e.g., *cro*, *z1466 (nanS-p), stx_2a_*, and *stx_2b_*) [97].

Su et al. used a Stx lysogen in *E. coli* strain MG1665 ΔMin27 (∆*stx::cat*) instead of a pathogenic STEC strain for transcriptomic analysis [127,130]. Their data differed from the one obtained with the original STEC strain. They observed upregulation of transport genes such as the flagellar synthesis genes *fliDESTZ* and acid resistance genes (e.g., *gadEW, hdeABD*, and *adiY*) [127].

A typical combination of EHEC-associated virulence factors is the production of Stx and the expression of a type three secretion system (T3SS) located on the LEE [131]. The acquisition of Stx phages, especially Stx2 phages, suppresses the expression of typical T3SS genes [44,97,128,129] (Figure 3). Xu et al. showed that strains carrying a Stx2 phage showed a decrease in *ler* expression, which is an important regulator of the LEE locus [129]. To further investigate a possible transcription factor responsible for this transcriptomic change, *cI*, *cII, cro*, *N*, and *Q* were cloned into a Stx2 phage lysogen of *E. coli* K12 and only CII showed a direct influence on T3SS expression [129]. Tozzoli et al. also identified a specific region between the *gam* and *cII* gene, which could encode one or more regulators that downregulate the T3SS, a characteristic which also points to a regulation involving *cII* [128].

Xu et al. proposed that the repression of T3SS by Stx phages provides the phage with complete control of this important colonization factor. They described a model in which expression of T3SS is controlled by the Stx phage and other effector-encoding prophages, allowing STEC to control the different regulatory elements depending on the stage of the infectious process [129].

Another study identified Cro as a regulator of T3SS during lysogeny under anaerobic conditions, and their findings were confirmed in a mouse model [95]. Cro seemed to activate 584 genes and repress 307 genes in the chromosome and, in particular, virulence factors such as fimbriae and flagella were upregulated by Cro.

The first study introducing RNA sequencing (RNA-Seq) to detect transcriptomic changes due to phage carriage showed that the *E. coli* strain MC1601 lysogenized with the φ24_B_ phage exhibited stronger expression of the GAD operon including the global regulator *gadE* and two other *gad* genes (*gadX* and *gadW*) [125] (Figure 3). These genes are responsible for the glutamate-dependent acid resistance mechanism, which is the most effective acid resistance mechanism in *E. coli* [132,133].

These data were in line with the study by Su et al. [127]. Furthermore, the role of the typical transcription factors CI, CII, and CIII was investigated, and found that CII might be involved in transcriptomic changes [125,128,129].

Veses-Garcia et al. hypothesized that there should be a differentiation between upregulated genes due to the SOS response and those due to phage regulation [125]. They observed an upregulation of DNA repair genes, iron, and phosphate acquisition and a downregulation of carbon, nitrogen, energy, and motility metabolism as well as a shift toward anaerobic respiration. Nevertheless, they found two phage-suppressed genes encoding for two pyruvate decarboxylases, which provide acetyl coenzyme A for the tricarboxylic acid cycle. These data hint at a downregulation of this metabolic pathway because acetyl coenzyme A is a key molecule [125].

Transcriptomic changes vary strongly under different environmental conditions. For example, Mitsunaka et al. observed a repression of *fliC* and *fliA* under anaerobic conditions in an *E. coli* K12 lysogen, which resulted in a repressed motility phenotype [134]. These results are in contrast to the study by Su et al., although it should be noted that though they both used K12 lysogens, the growth conditions differed [127]. Therefore, it is necessary to add phenotypic experiments to verify the transcriptomic data. Additionally, the heterogeneity of Stx phages may also be responsible for different transcriptomic changes [126]. Hence, the comparison of diverse studies has to be done carefully.

Berger et al. also used RNA-Seq to analyze transcriptomic changes comparing two different *E. coli* K12 strain MG1655 lysogens [126]. One of the lysogens carried the Stx2a phage (φO104) of the hypervirulent outbreak strain *E. coli* O104:H4, which caused a massive disease outbreak in Germany in 2011 [135]. The closest related phage is the PA8 phage of pathogenic STEC serotype O157:H7, and it was therefore chosen as the second phage for lysogenizing the same strain [126]. The transcriptomic changes in this study were not under SOS response-inducing conditions. Upregulated genes were mainly sulfur-, motility, and chemotaxis-related. Additionally, some genes of the SOS response and several metabolic genes for mixed acid fermentation were upregulated. Most downregulated genes were involved in carbon source transport and metabolism. CI and Cro were not found to be responsible for the detected transcriptomic changes. The results of the study allowed the authors to suggest that φO104 and φPA8 changed the metabolism of the host significantly and that both phages might provide the host with more fitness under in vivo conditions [126].

Other studies identified prophage-encoded small regulatory RNAs (sRNA), which regulated gene expression on the chromosome, as, for example, Esr41/EcOnc 14 from the *E. coli* Sakai strain [44]. Waters et al. found three mRNA interaction partners for Esr41: *cirA*, an iron siderophore complex uptake receptor; *bfr*, bacterioferritin; and *chuA*, an outer membrane heme receptor. Esr41 binds in all three cases at the ribosome binding site, which suggests a translation inhibition [136].

Tree et al. investigated prophage-encoded sRNAs such as AsxR and AgvB, designated “sponge” RNA, in pathogenic STEC. They bind to the RNA-binding protein Hfq and function as “sponges” or “anti-sRNA” for the chromosomally expressed sRNAs FnrS and GcvB, respectively [44] (Figure 3).

Other studies identified prophage-encoded small regulatory RNAs (sRNA) that regulate gene expression on the chromosome. One of these is Esr41/EcOnc 14 from the EHEC Sakai strain [44]. Initially, this sRNA was hypothesized to enhance flagellin expression [137].

Another example of transcriptomic change via phage-encoded sRNAs is the IpeX sRNA encoded on the Stx-producing phage φPA2. The expression of IpeX reduces the expression OmpC and OmpF (outer membrane porins) [138,139]. The last example is also encoded on Stx-producing phage φ24_B_. The function of 24b_1 sRNA is elusive but might mimic eukaryotic microRNAs [140]. The deletion of this gene encoding 24B_1 leads to better prophage induction, enhanced phage production, and different bacterial cell adsorption capabilities [141].

It is clear that Stx phages have a remarkable impact on the host transcriptome. Most studies agree that these phages provide the host with increased acid resistance [125,127] and motility [126,127]. On the other hand, they also seem to be responsible for repression of LEE1 [128,129] and metabolic pathways involved in energy metabolism [97,128,129], fatty acid metabolism [97,127], carbon source utilization [125,126] (Figure 3), and directly or indirectly in the tricarboxylic acid cycle (TCA) cycle [125,126,127]. Summing up, this also makes the Stx-encoding phage a potential metabolic burden [127]. The function of many genes in Stx-encoding phages, but also in other prophages of STEC, are still elusive [9]. Ongoing research will most probably reveal even more transcriptomic changes upon Stx phage carriage since we have only begun to understand the function of some of the genes encoded in Stx phages. This is the case for phage φ24-B, which possesses five conserved genes within the *exo*-*xis* region; *ea22*, orf60a, orf61, orf63, and orf73 [142,143,144,145]. *ea22* and orf73 promote the maintenance of the lysogenic state, orf63 represses and delays phage induction, while orf60 and orf61 seem to promote phage induction since their deletions significantly delay the induction of the Stx φ24B prophage [142,143,144,145,146]. Moreover, adsorption of phage φ24B on *Escherichia coli* host cells was shown to be less efficient in the absence of either orf60a or orf61 [142].

## 8. Impact of Stx Phages for the Human Host (Impact on Enterocytes and Immune System)

The environmental conditions of the human body have an effect on Stx-phage induction and consequently on the expression of Stx by the bacterial hosts [147]. At the same time, the toxin has an effect on the human (or animal) body that ranges from the cytopathic effect in different cells to interaction with blood components [148,149]. However, it is unclear what the pathogenic potential of the Stx phages is once released from the cell, particularly if they can play a role other than serving as mere vehicles for the toxin gene. It is known that phages can induce the immune system response [150,151] and, as a part of this response, they can stimulate phagocytosis. One question arises then, as to whether Stx phages can directly interact with the human (or animal) cells, be phagocyted, and have an effect beyond their role in STEC pathogenicity. Bentancor et al. demonstrated that the “prokaryotic” Stx2 sequence, when introduced in eukaryotic cells, potentially allowed the expression of Stx. This expression was assessed by the activity of the toxin, by the generation of anti-Stx antibody responses, and because it caused mortality in mice [152,153]. Moreover, the lysogenization of the laboratory strain *E. coli* C600 by Stx phage 933W was sufficient to cause renal and intestinal damage in a mouse model in the absence of other STEC pathogenicity factors [154]. This damage could be caused by bacterial Stx expression in the absence of other adhesion or invasion factors, by the transduction of Stx phages to other bacterial hosts in the gut or, considering previous studies, by direct interactions of free Stx phage virions with the eukaryotic cells. Although this last option was not confirmed, free 933W phages were detected in the brain tissue of mice, in the same areas where Stx activity, astrocyte activity, and neuronal damage were detected [154]. Phages can translocate more effectively than bacteria along the human tissues [150,155], and they are abundantly found in different human samples such as ascitic fluid, blood/serum, urine, or cerebrospinal fluid [156].

Regardless of the role of Stx phages, the expression of Stx may modulate the innate immune response of human enterocytes. STEC(EHEC)-derived Stx inhibits NF-κB signaling and chemokine gene expression in T84 cells [157]. Moreover, observations indicate that strains expressing Stx1 and Stx2 produced fewer chemokines than the isolates only harboring one type (stx2) [158].

Stx also favors the attachment of the bacteria to the colonic epithelium. Enhanced colonization of O157:H7 expressing Stx has also been demonstrated and attributed to an Stx-mediated increase of nucleolin, an eukaryotic receptor recognized by the intimin, responsible for the intimate adhesion of the pathogen to the enterocyte surface [159]. However, these observations were attributable to the toxin expression and there is no direct evidence of the role of Stx phages on adherence other than the modulated level of Stx expression [159].

When observing the impact of Stx phages in animal hosts, it has been observed that Stx1 markedly induced apoptosis in a stimulated B lymphoma bovine cell line, while hindered the proliferation of bovine lymphocytes by blocking their activation and, consequently, causing suppression of the mucosa-associated immune response against STEC infection [160]. Again, the effect was attributed to the Stx1 activity, and not to the phages themselves.

Stx2, particularly Stx2a and Stx2c subtypes, affect regeneration of the gastrointestinal epithelium in calves. Higher STEC transmission and excretion levels from the animals colonized by strains expressing the Stx2a subtype has been shown since Stx2a enhances *E. coli* O157 colonization of calves by restricting regeneration and turnover of the colonized epithelium [119]. Here, the role of Stx phages was confirmed, as a faster induction of gene expression from the Stx2a-encoding prophage compared to that from the Stx2c-encoding prophage can account for the more evident effect of this toxin subtype. In contrast, this same study did not show evidence to support a role of Stx2a in immune suppression [119].

## 9. Structure and Function of Non-Stx Phages of Pathogenic STEC

Aside from Stx phages, other non-Stx prophages are found in the genome of STEC, some of them including complete and inducible phages, but also non-inducible, remnant, cryptic, or residual phages. Polylysogeny is therefore a very common occurrence in STEC strains, and a good example is O157 strain Sakai, which carries up to 18 different prophages [13]. As temperate phages, prophages preferentially belong to the *Siphoviridae* or *Podoviridae* morphological types [161] and usually display a modular structure, the so-called genetic mosaicism [162]. Similar sequences are also shared by different phages. For this reason, it is difficult to distinguish between Stx and non-Stx phages in the STEC complete genomes because these similar sequences confound the software used for contigs assembly, producing false chimeras. This problem is overcome when using sequencing platforms that generate longer reads [47], or by inducing and isolating the prophages before sequencing [10].

Nevertheless, the abundance of prophages in STEC strains suggests some advantage for the actors implicated, that is, bacteria and phages. Bacteria seem to keep all this prophage pool to incorporate new genetic traits [163], but also to enhance the mobilization of their genome [13,164] or, as mentioned in the previous section, confer fitness and improve growth, or regulate other elements.

Prophages coexisting in a bacterial genome also take advantage of polylysogeny, increasing their genetic diversity. Multiple recombination events between prophages located in the same genome might occur [16,163], mainly between the identical fragments of DNA shared by the co-existing prophages. These shared sequences serve to anchor the activity of recombinases, which in many cases are encoded by the prophage genomes themselves [165] or that can be provided by the host. For example, new Stx1 phages are generated after recombination events occurring between the Stx1 and Stx2 prophages [13].

Other genetic elements can interact with prophages, for example, by taking their capsids to mobilize themselves; in *E. coli*, this fact has been described for genomic islands [166], defective prophages [14,167,168], and plasmids [169].

## 10. Interaction between Stx and Non-Stx Phages

Stx phages are the most studied phages because of their implication in STEC pathogenicity, but there are other prophages related with STEC virulence such as the temperate phages encoding different types of toxins or phages encoding effector proteins (Table 1).

Not only temperate phages contribute to the repertoire of weapons used by STEC. Virulent phages, which mostly conduct generalized transduction, might provide STEC with antibiotic resistance. While specialized transduction is the canonical mechanism for incorporating genes through temperate phages that harbor a single and specific gene in a precise location of their genome, generalized transduction is understood as the mechanism that mobilizes any fragment of the bacterial genome [189]. The bacterial DNA is cleaved at a specific sequence (*pac*-like site), which is similar to the *pac* site located in the phage DNA from which phages start filling their capsids. This “mistake” leads to phage capsids full of bacterial DNA instead of phage DNA. Generalized transducing particles are not competent phages since they cannot propagate, but they can transfer any fragment of bacterial DNA including genes of interest involved in the bacterial pathogenicity such as antibiotic resistance genes (ARGs) [190]. *E. coli* species including pathogenic *E. coli* use their arsenal of prophages to mobilize specific genes by specialized transduction, but they could also mobilize other DNA fragments including ARGs [191]. The origin of ARG-transducing phage particles is thought to be the bacterial strains, but they can also be found as free particles in different environments [181,192] (Table 1).

Although generalized transducing particles are a likely source of ARGs detected in phage particles, there are some studies in which ARGs have been detected in phage particles able to propagate in a host strain, for instance, in *E. coli* K-12 derivatives (Table 1). Since generalized transducing phages should not be able to infect and propagate using a host strain, it is assumed that other phage particles, virulent, or temperate are responsible for the new progeny of phages encoding the ARG. Although the nature of the ARG-encoding phage particles detected is not known, it is assumed that both specialized and generalized phage particles contribute to the transmission of ARGs originated in the chromosome or the plasmids of the host strains, and that all contribute to the pool of phages that can be detected in numerous environments.

The third newly described transduction mechanism, the lateral transduction [193], involves prophages that, by delaying the excision from the bacterial chromosome, can package enormous amounts of bacterial DNA. This mechanism provides a better explanation than the generalized transduction for the high amounts of ARG-carrying phage particles observed after induction of clinical *E. coli* strains [191,194]. Although it was initially described in *Staphylococcus*, it is reasonable to believe that similar or the same mechanism will be reported soon in *E. coli*.

In addition to non-Stx phages carrying genes directly related to virulence that are incorporated in STEC by transduction, the presence of other non-Stx prophages have a direct influence on the STEC pangenome and its pathogenicity. As above-mentioned, Xu et al. proposed a model in which expression of T3SS is controlled by the Stx phage, but also by non-Stx phages encoding T3 effector molecules [129], or Lom or Bor genes in prophages confer serum resistance and enhance adhesion (Table 1).

Prophages present in an STEC genome are used as protection against superinfection by other phages. Non-lysogens are vulnerable to phage infection and lysis while the lysogens are immune to superinfection with certain phages. For instance, *E. coli* prophage Qin, also found in STEC, is a cryptic prophage that encodes a small protein, DicB, which protects the lysogens from future phage infections [195].

Several physiological functions such as stress tolerance, biofilm formation, antibiotic resistance, and other advantages to the host genome are attributed to prophages in *E. coli* (Wang et al. 2010) [196]. For example prophage e14, present in different *E. coli* strains, encodes important functional genes such as *lit* (phage T4 exclusion), *mcrA* (modified cytosine restriction activity), and *pin* (recombinase) [197]. A study conducted by deleting nine non-Stx cryptic prophages (CP4-6, DLP12, e14, rac, Qin, CP4-44, CPS-53, CPZ-55, and CP4-57) from the *E. coli* genome concluded that different prophages protected the lysogen against different antibiotics against other environmental stresses such as acid, heat, osmotic and oxidative stresses, and increased biofilm formation, essentially by inhibiting cell division [196].

Although not related to the STEC-typical mechanisms of pathogenicity, induction of *E. coli* prophages suggested an indirect role of the bacterial phages in the modulation of human host immunity and in a particular case, this was associated with the development of type 1 diabetes [198]. Induction of *E. coli* prophages causes depletion of the population of amyloid-producing *E. coli*. Many pathogenic *E. coli* produce curli, which are a type of naturally-occurring amyloid fibers. Depletion of *E. coli* populations producing curli has been associated with seroconversion, a period during which autoantibodies to antigens of pancreatic β-cells or insulin are produced, finally leading to the development of diabetes type 1 in children [198].

In contrast, certain prophages are inserted into transcriptional factor *mlrA (yehV)*, a regulator responsible for curli generation and biofilm formation, therefore in situations in which prophages are inserted, curli and biofilm production in some *E. coli* O157:H7 isolates are abolished, reducing bacterial pathogenesis [199].

## 11. Conclusions

Many years of phage research in the field of pathogenic STEC have shed light onto diverse genetic features of their lambdoid phages and have helped to better understand the manifold functions hidden behind these heterogeneous particles. Nevertheless, a series of questions remain unresolved and, therefore, Stx phage research represents an important tool to better elucidate the distribution of phages in their hosts, their contribution to the bacterial metabolism, and finally, to the development of human disease. This knowledge will only be possible with approaches that include strong interaction among different scientific disciplines (e.g., medicine, microbiology, genetics, biochemistry, biophysics, bioinformatics, and biotechnology).

## Figures and Tables

**Table 1:** Virulence factors other than *stx* and antibiotic resistance genes (ARG) encoded in the genome of *E. coli* phages.

Source of Phage	Virulence Factor or ARG	*E. coli* Strain *	Phage	Phage Replication	Reference
Induced from bacteria	Toxin: Cytolethal distending toxin I	Enteropathogenic *E. coli* O127:H7	CDT-1φ	Temperate	[170]
Induced from bacteria	Toxin: Cytolethal distending toxin V	Enterohemorrhagic *E. coli* O157:H7	φ125	Temperate	[171]
		Environmental *E. coli* O22:H8	φ62		
Sequenced from bacteria	Toxin: Cytolethal distending toxin V	Enterohemorrhagic *E. coli* O157:H43	P2-like	Non-inducible, temperate	[172]
Induced from bacteria	Adhesion: Lom	Diverse *E. coli* strains	Various lambdoid phages	Temperate	[173]
Induced from bacteria	Serum resistance: Bor	Diverse *E. coli* strains	Various lambdoid phages	Temperate	[173]
Induced from bacteria	Immunoglobulin binding protein: EibD	*E. coli* reference strain ECOR-9		Temperate	[174]
Induced from bacteria	T3SS effector protein: Cif	Enteropathogenic *E. coli* O103:H2	Cif-encoding phage	Temperate	[175]
Sequenced from bacteria	T3SS effector protein: EspF (TccP)	Enterohemorrhagic *E. coli* O157:H7 Sakai	CP-933U	Cryptic prophage	[176]
Sequenced from bacteria	T3SS effector protein: EspJ	Enterohemorrhagic *E. coli* O157:H7 Sakai	CP-933U	Cryptic prophage	[176]
Sequenced from bacteria	T3SS effector protein: EspL2	Enterohemorrhagic *E. coli* O157:H7 Sakai	SpLE3 phage-like element	Unknown	[177]
Induced from bacteria	T3SS effector protein: NleA	Attaching-effacing *E. coli* O84:H4	Stx1 phage	Temperate	[51]
Induced from bacteria	T3SS effector protein: NleB, NleC, NleH, NleG espJ, and nleA/espI	Enteropathogenic *E. coli* Enterohemorrhagic *E. coli*	Cif-encoding phage	Temperate	[175]
Sequenced from bacteria	T3SS effector protein: NleC	Enterohemorrhagic *E. coli* O157:H7	CP-933K	Cryptic prophage	[178]
Sequenced from bacteria	T3SS effector protein: NleD	Enterohemorrhagic *E. coli* O157:H7	CP-933K	Cryptic prophage	[178]
Induced from bacteria	T3SS effector protein: NleH	Enteropathogenic *E. coli* O127:H7		Temperate	[170]
Induced from bacteria	Metabolism: NanS-p, *O*-acetyl esterase for the use of sialic acids	Enterohemorrhagic *E. coli* O157:H7	Various phages	Temperate	[102]
Sequenced from bacteria	ARG: *aadA1*, *aadA2*, *aadA22*, *aac(3)-Via* and *APH(4)-Iabla*_TEM_,*cmlA1*, *drfA*, *mefB sul1*, *sul3*, and *tet*(C)	Enterotoxigenic *E. coli* (ETEC)	Various phages	Temperate	[179]
Isolated from vegetables	ARG: *bla*_CTX-M1_, *bla*_CTX-M9_, *bla*_VIM_, *mecA*, *armA*, *qnrA*, *sul1*	Replicative on non-pathogenic *E. coli* WG5 strain	Phage pool	Unknown	[180]
Isolated from soil	ARG: *bla*_TEM_, *bla*_CTX-M1_, *bla*_CTX-M9_, *armA*, *qnrA*, *qnrS*, *sul1*	Replicative on non-pathogenic *E. coli* WG5 strain	Phage pool	Unknown	[180]
Isolated from meat	ARG: *bla*_TEM_, *bla*_CTX-M1_, *bla*_CTX-M9_, *bla*_OXA-48_*, bla*_VIM_, *armA*, *qnrA*, *qnrS*, *sul1*	Replicative on non-pathogenic *E. coli* WG5 strain	Phage pool	Unknown	[181]
Isolated from chicken meat	ARG: *aphA1*, *bla*_TEM,_*floR*, *tetA*	Replicative on non-pathogenic *E. coli* DSM 12242 strain	Various phages	Virulent	[182]
Isolated from fish	ARG: *bla*_TEM_, *bla*_CTX-M1_, *bla*_CTX-M9_, *bla*_OXA-48_*, bla*_VIM_, *armA*, *qnrA*, *tetW*, *sul1*	Replicative on non-pathogenic *E. Coli* WG5 strain	Phage pool	Unknown	[183]
Isolated from dog urine	ARG: *bla*_TEM_, *sulI*, *sulII*, or *strA*	Replicative on non-pathogenic *E. coli* DSM 12242 strain	Various phages	Virulent	[184]
Isolated from surfaces of equine veterinary clinics	ARG: *addA1, cmlA*	Replicative on non-pathogenic *E. coli* DSM 12242 strain	Various phages	Virulent	[185]
Isolated from wastewater	ARG: *Fragments of bla*_TEM_, *cmlA*, *qnrS*, *tetM*, *mcr-1 sul 3*, and *sul4*)	Replicative on various multidrug-resistant *E. coli* hosts	Two T7-Like phages	Virulent	[186]
Isolated from soil and water	ARG: *bla_CTX-M_*	Replicative on *bla_CTX-M_*—negative environmental *E. coli* isolates	Seven T4-Like phages	Virulent	[187]
Induced from bacteria	ARG: *bla_SHV-2_*	Clinical *E. coli* O8:H19	P1-like phage RCS47	Temperate plasmid-like	[188]

* Definitions of pathogenic *E. coli* are according to Nataro and Kaper [131]; ARG: antibiotic resistance genes; T3SS: type III secretion system.
